# Influence of the Leucocyte Count in the Decision for or against Immediate Operation1Read at the 36th annual meeting of the Medical Association of Central New York, held at Auburn, September 22, 1903.

**Published:** 1904-01

**Authors:** E. S. Van Duyn

**Affiliations:** Syracuse, N. V.; Lecturer in surgery, Medical College Syracuse University; surgeon Hospital of the Good Shepherd.


### Influence of the Leucocyte Count in the Decision For or Against Immediate Operation.[1]

E. S. VAN DUYN, M. D., Syracuse, N. Y.

Lecturer in surgery, Medical College Syracuse University; surgeon Hospital of the Good Shepherd.

FOR the illustrative purposes of this paper, fifty consecutive cases have been tabulated in which a leucocyte count was made prior to operation or at the time when the question of operation arose. The counts were made with reference to the additional information that could thus be gained as to the nature of the case, the presence or absence of infection, its character and severity. In dealing with surgical conditions, we know that when toxic material is being absorbed by the system, there is a polymorphonuclear leucocytosis present in the blood, and that when the material being thus absorbed is pyogenic in origin this increase of leucocytes in the blood is marked, its degree depending on the nature of the infection, the amount of material being absorbed, and upon the vitality or reaction of the patient. Further, that when the intoxication progresses and the severity of the case is increasing, there is present a proportionate increase in the amount of leucocytosis.

Basing our deductions on this principle, our endeavor was to determine by the leucocyte count, the presence of infection, the severity of its character, its extent and progress, and so decide as to the necessity of operation, its urgency and somewhat as to the prognosis. Many times the conditions found and the results obtained in these cases were proportionate with the amount of leucocytosis present, but in many of the cases, at first sight, there seem to be a wide variation. Apparently when this factor was alone considered these exceptions were so frequent that no great reliance could be placed on the deductions thus made.

A careful consideration of the many conditions that influence the presence and amount of leucocytosis is essential to a correct understanding of the meaning of the count. Only when all the conditions that affect the number of leucocytes are recognised and

1. Read at the 36th annual meeting of the Medical Association of Central New York, held at Auburn, September 22, 1903.

proper allowance made for their influence, does the leucocyte count become of value as a guide. As these conditions are more carefully studied and considered and their influence becomes known and appreciated, the more constant and accurate becomes the leucocyte count as a basis on which trustworthy deductions may be made as to the presence, severity and character of any suspected septic process present. Care was taken to have the count accurate and made at as suitable a time as possible to exclude other conditions. Active digestion increases leucocytosis and an alimentary intoxication from disordered digestion often greatly increases the polymorphonuclear elements. Such states as fatigue, nervousness and fear, diminish the leucocyte count.

In one of these cases reported, a count of 19,000 fell to 6,000 during the progress of the chill and a single count taken at that time, if not correctly understood, would have been entirely misleading. Again, a leucocytosis is present in pregnancy, as is also illustrated in one of these cases. Moreover, those constitutional diseases which are associated with an increased leucocyte count must be recognised if present and due allowance made for their effect. When time permitted, repeated counts were made a few hours apart. The stationary or increasing character of the count obtained by such observations showed a slow or rapid progress of the trouble, and in doubtful cases, when not increasing a moderate count encouraged delay in operating until the nature of the case was better deducible from longer observation and further development of the symptoms. A rapidly increasing leucocytosis showed the presence of a progressing intoxication and indicated, where operation was in question, its early performance. In tabulating these cases, we noted, first, the symptoms, temperature, duration and general condition; second, the diagnosis as made from them; third, the amount of leucocytosis present; fourth, the deductions made therefrom; fifth, the operative or other procedures adopted; and sixth, the actual condition discovered by the operation, or as shown by the course of the subsequent events,

In these fifty cases, the lowest count was 5,600 in an anemic child with incipient tuberculosis involving the knee joint, and the highest was 37,000 in an acute appendicitis with peritonitis. Twenty of these cases showed no pus infection, while thirty did. In these first twenty, the count ranged from 5,600 to 14,600, but in eleven of them, the count was below 9,400, while in the nine others, the higher count without pus infection was explained by the other conditions present. These were as follows: one with a leucocyte count of 12,000 was a very acute case of catarrhal appendicitis, the appendix greatly congested and swollen, with a temperature from 101° to 102°, the symptoms of the case increasing in severity. The second with a count of 12,500, also was appendicitis of only forty hours' duration, in which no pus was found, yet the culture made from the contents of the removed appendix, gave a culture of streptococcus pyogenes aureus. The third, with a leucocyte count of 12,500 was that of a patient whose condition was one of marked general malnutrition. The count increased after operation, and the patient died a week later, the wound showing a gangrenous condition without the slightest evidence of any healing process having been established. The fourth with 14,600 leucocytes, was complicated with pregnancy and in all the remaining cases, without pus and above 9,000, malignant disease was found.

In the thirty cases in which pus infection was found the count ranged from 7,000 to 37,000, but here again the acute infectious cases uniformly conformed with the rule given above, and gave a high count above 15,000. Of these cases below 15,000 with pus present, five gave a count below 9,000 here, three were tubercular, and two gave a pure culture of bacillus coli communis. In the cases between 9,000 and 14,000, nine in number, eight were cases of chronic abscess, or chronic pus tube, in which the symptoms were subacute and mild in character and the pus was found tubercular or gonorrheal in type, or confined to an abscess cavity, the walls so thickened and covered with pyogenic membrane that there was little absorption. The one case below 15,000 in which acute pus formation was found was a case of acute appendicitis in which the appendix was found ruptured and a small mass of thick yellow pus had been exuded. This pus laid directly at the point of rupture and was a dense circumscribed mass. The appendix was nowhere gangrenous and the wound closed entirely in less than a week. Here, from the condition found and the symptoms present, there seems no question that the rupture occurred just prior to the operation, or was caused by its manipulations. The leucocytosis present in this case was 11,600.

In these fifty cases then, twenty of them with a leucocyte count ranging from 5,000 to 15,000 with no pus infection present, and thirty, with a range between 7,000 and 37,000, with pus infection, there is an overlapping of forty of the fifty cases, or 80 per cent. of the total number. At first glance this would seem to preclude the possibility of judging by the leucocyte count as to the presence or absence of an infectious process; but when we subdivide them in this way and recognise the influence of the concurrent conditions, and take into account all that can be learnt from the other symptoms present, as to the probable nature of the infection, the duration and progress of the disease, we find under stated conditions the amount of leucocytosis quite constant within narrow limits and positive as to its indication. Conclusions deduced in this way, become of value, and as the influence of all the conditions that modify the amount of leucocytosis are better understood, it becomes a more and more reliable indication of the presence or absence of an active infective process.

The points further deducible from the leucocyte count in these cases than the simple presence or absence of an acute infective process are best shown by a brief recital of illustrative cases.

Case I.—Male, 50 years old, general condition good, taken with sudden pain in epigastrium, vomiting and chill. Temperature 100°. History of previous similar attacks with pain always to the left of midline. On the third day of present attack, the pain became more severe and for the first time located in the region of the gall-bladder. Tenderness and increased resistance was present. Gall-duct obstruction was diagnosticated. A leucocyte count of 14,000 was found and a beginning pus infection was diagnosticated. Immediate operation was advised and performed. The gall-bladder was found much thickened and the tissues surrounding it were firmly united with it by newly formed adhesions. The gall-bladder itself was distended by a thin, bloody pus, which was evacuated and the cavity drained. Four days later the leucocyte count, which had fallen, rose to 19,000 and, on investigation, a small pocket containing a greenish yellow pus was found in the lower end of the abdominal wound. The leucocytosis immediately fell on its evacuation and drainage. In comparison with this, I cite a second case.

Case II.—A woman, 68 years of age, with symptoms similar to those in the first case, excepting that they were less acute; there was marked jaundice, and the temperature did not rise above 99.5°. In this case the leucocyte count was only 6,000. Here no infection was considered present and this was borne out by the condition found on operation. The gall-bladder was thin, movable and contained a thin, clear gall and many stones, but no pus.

The next two cases, both of appendicitis, show, to a still greater degree, the same difference in the nature and degree of infection and the necessity of immediate operation. The first of these was a male 36 years of age, general condition good, had been suffering for ten days with the symptoms of a mild attack of appendicitis. The temperature never rose more than one degree above normal. On the tenth day the leucocyte count was 12,000. Operation disclosed a catarrhal congested condition of the appendix without pus formation. The second case, also a male, 16 years old, was seen first on the seventh day of attack. His temperature had fallen from 102° to 99°, and the patient was in an extremely apathetic condition. His leucocyte count was 37,000. Operation disclosed two distinct pus cavities and drainage was left. Following the operation, however, the symptoms continued severe, leucocytosis only fell, 36 hours after operation, to 33,000 indicating the continued activity of the infectious process and an unfavorable prognosis. The wound showed an unhealthy appearance, the symptoms continued severe, fecal vomiting set in, and on the fifth day after operation the patient died.

In the first of these two cases, the low leucocyte count showed the absence of any marked degree of inflammation or pus infection and corroborated the previous diagnosis, verified by the operation, of a catarrhal inflammation of the appendix. In the second case, the high count showed clearly the severe nature of the infection, and that the fall of temperature and diminution of pain before operation, were due to shock and exhaustion, but not to an improvement in the condition. In both of these cases, immediate operation was indicated, but in the first, a good outcome was to be expected, while in the second, the unfavorable result obtained was only too clearly foretold.

When the infection is purely of a tubercular nature, there is a low leucocyte count, however severe the symptoms. This is well shown in the record of a ward patient, a male, 27 years old, who was sent in with a previous diagnosis of delirium tremens. His temperature ran from 100° to 103°. His urine contained much pus, many fine granular casts, and some blood. There was great tenderness and some bogginess over the area of the left kidney posteriorly, and the area of dulness was enlarged. Nephrectony was performed. The kidney was found enlarged, lobulated and it contained many pockets of pus. The leucocyte count before operation and after was only 9,000, yet a week later he died of a general tubercular infection involving the meninges, both lungs and the peritoneum.

There were six cases in which the leucocyte count assisted greatly in making a differential diagnosis and prevented operation, though this course was strongly urged. The first, a female, married, 24 years old, was taken with sudden severe abdominal pains and tenderness, at first general, then localised above the pubes. The next morning, temperature reached 104.4°, the tenderness was more on the right side, and a slight chill preceded the rising temperature. These symptoms, with a negative Widal reaction, were considered to point to appendicitis. Two leucocyte counts taken several hours apart, showed only 7,600 and not increasing. On account of this lack of any leucocytosis, it seemed perfectly safe and better to wait the further development of the symptoms. On the fourth day the spleen showed enlargement, on the fifth, a profuse roseola developed, and on the sixth a positive Widal reaction for the typhoid bacillus was obtained. In a second case of typhoid, also in a young lady 16 years old, with unusually severe abdominal symptoms, and an equally atypical temperature chart, the correct diagnosis was early made on a repeated normal leucocyte count, though the roseola was absent, and the Widal reaction remained negative until the tenth day.

The leucocyte count was of equal assistance and a protection to the patient in yet another case. Surgical interference was sought in a case of a young woman, 17 years old, the symptoms beginning abruptly with slight chill, temperature 101.2°, vomiting and severe abdominal pains and tenderness in the right iliac region. These symptoms gradually subsided, but ten days later they all returned with increased severity. Appendicitis was diagnosticated and operation was urged, but the leucocyte count showed but 9,000, and an expectant plan of treatment was advised and followed. The menstrual flow began the next day, all the symptoms subsided and the patient's recovery has been permanent. Another case, seen in consultation, was that of a child 10 years old, with acute knee-joint trouble of five weeks duration. The temperature had been running a continuously elevated course of from 100° to 102.4°; pulse small and rapid, child pale, thin, and emaciated, knee swollen, red, hot, painful and tender. A leucocyte count of only 5,000 showed no staphylococcic or streptococcic infection to be present, though the result of traumatism. Fixation and extension were advised, rather than any operative procedure to open the joint, and under this treatment the active symptoms quickly subsided. The fourth of these cases, a female, 24 years old, single, who had had trouble with a movable kidney for some years, was seized with sudden severe pain in her right side, and the question of infection arose. A count of 8,750 seemed to indicate no absorption of toxic substances, and under simple rest in bed the acute symptoms were relieved. The extremely poor general condition of the patient at the time operation was proposed, argued strongly against its probable success, and this procedure was fortunately contraindicated by the leucocyte count.

The next was the case of a girl 19 years, single, in bed eight weeks, with traumatic hysteria following a slight blow on the head. Much pain in the back was complained of, especially on the right side and radiating into the right leg. A sudden development of the presence of albumin and pus in the urine, with some granular casts, and scanty flow was diagnosticated as abscess of the kidney, and the cause of the continued pain that has existed on that side, was thought to be found. The leucocyte count remained at 7,000 to 7,200, operation was delayed and in four days the urine entirely cleared and since then has remained clear.

In the last of these six cases, a female, 29 years old, delivered three days previously of a child, suddenly developed an intense painful swelling of the left parotid gland, the temperature ranging from 102.5° to 104.8°. Abscess from infective embolus was diagnosticated, but as the leucocytosis did not rise above 8,000 incision was delayed, though strongly advised, and in four days all the symptoms subsided.

In the next two cases the leucocyte count was of the greatest assistance in indicating the amount of septic intoxication present at the time of operation, and so influencing the method employed as well as the prognosis after operation. A young man, 24 years of age, was operated on for intestinal obstruction. A gangrenous Meckel's diverticulum was found ruptured. Though its appearance was that of a recent occurrence, it was impossible to tell whether the rupture had occurred sometime previous to the operation, or just before, or during its performance. The leucocyte count taken just previous to the operation was only 9,000, in spite of the severity of the other symptoms present. The gangrenous area was excised, the intestine united with Lembert sutures and the abdominal incision closed without drainage. The conclusion drawn from the count of 9,000 that there was no considerable peritoneal infection present at the time of operation, was substantiated by the uncomplicated recovery of the patient.

The other of these cases, was that of a boy, 13 years old, who had been suffering ten days with what his physicians styled "a walking appendicitis." At the end of that time the abdomen became tense, and the pain increased and became general; temperature, 101°. Immediate operation was performed. The abdomen was found distended with a considerable quantity of thick, milky fluid, the appendix ruptured and bathed in a thicker pus. The leucocyte count taken just previous to operation, was 7,200 and in connection with a seeming general good condition of the patient, in spite of the severity of the condition found, a favorable prognosis was given. The fluid from the cavity and the pus from the appendix gave a pure culture of bacillus coli communis. This prognosis was verified by the recovery of the patient.

The last two cases are equally instructive as to the part the leucocyte count played in determining the necessity of immediate operation and the method to be employed. The first, a female, 33 years old, gave a history of an abortion a year before, following which she had suffered from an almost continuous uterine hemorrhage lasting five months, which left her in a very weak and emaciated condition. For the last three months she had constant increasing abdominal pain. On entrance to the hospital her temperature was 102°, pulse 120, and the clinical picture was one of pus infection. So great was the exhaustion and so poor her general condition, any operative procedure would necessarily have been attended with the greatest risk. It was decided, if possible, to delay the operation and first improve her general condition. The leucocyte count was found to be but 11,600 and not increasing. Rest and stimulants for ten days improved her general condition quite appreciatively, but at the end of that time she had a chill, temperature rose to 104°, and the leucocyte count increased to 14,000. Operation disclosed the pelvic organs and lower bowel matted together in a mass of adhesion, and at the location of the left tube there was a mass to a large extent solid, but containing a small cavity of greenish pus that was ruptured during removal of the tumor, necessitating the use of abdominal drainage. The pathological report indicated that the tumor was a myxosarcoma. Although the condition of the patient after the operation was extremely low, the blood count showed a decrease of 2,000 and gave encouragement. Her gain, though slow, was continuous and at the end of six weeks she was able to leave the hospital. If operation had been performed at the time of entrance into the hospital, it is probable she would not have survived its performance, owing to her extremely feeble resistance.

In contradistinction to this case I will cite one more, in which the rapidly increasing and already high leucocyte count hurried me to immediate operation, which disclosed a condition the seriousness of which was not shown by the other symptoms present. Mrs. ———, 50 years old, previously well, was suddenly seized with a diffuse abdominal pain followed by vomiting and marked distention of the abdomen. Her bowels, however, moved freely. She was admitted to the hospital on the next afternoon with a temperature of 101.8°, pulse 100. Her general condition was seemingly very good. Her mind was clear and there was little nervousness or shock. Bimanual examination under cholorform disclosed no tumor or mass and was otherwise negative. At 5 p. m. the leucocyte count was 18,000; four hours later, it increased to 20,000, whereupon I did laparatomy. On cutting through the abdominal peritoneum a thick, badly smelling, greenish fluid gushed out in considerable quantity from the free abdominal cavity. A ruptured pus tube was found on the right side. Complete hysterectomy was performed and free drainage was obtained by the vagina. Her general condition continued good, and the following day the leucocyte count had decreased 2,000. On the next following day it had decreased to 13,000 and twenty-four hours later, had fallen to 9,000. Ten days later the leucocyte count suddenly increased to 15,000. A small mural abscess was found, which was evacuated and drained, following which the count dropped again. The patient left the hospital three weeks later apparently well, all discharge having entirely ceased.

These cases of themselves are far too few from which to draw any rules to govern us in estimating the value of the leucocyte count as an aid in the question of the employment of operative procedures. They offer many suggestions however, and as they bear out the conclusions of others, based on extended statistics, and show the correctness of their deductions, they are both interesting and valuable. It is not the making of fixed rules between the grades of leucocytosis and any special pathological surgical condition that is of importance, but due allowance being made for the influence of possible concurrent conditions, it can be said in this class of cases first, that with a normal leucocyte count there is no pyogenic intoxication or active pus formation present; second, that a high degree of leucocytosis is a positive indication of the presence of a septic process; third, that an increasing count shows the severity of the condition present is increasing and indicates immediate operation; and fourth, a stationary moderate degree of leucocytosis allows delay in the employment of operative procedures. I should say that the line that separates a high count from a low one falls between 10,000 and 15,000.

With a leucocyte count above this line, other conditions permitting, in this class of cases, I should always advise immediate operation, however mild the other symptoms; especially would this be so when the repeated counts had shown an increasing leucocytosis. But when the other symptoms pointed strongly to the apparent necessity of operation, a low leucocyte count should not deter its immediate employment. While great assistance can be gained from the leucocyte count, the knowledge to be obtained from all the other signs and symptoms present lose none of their weight and importance and our conclusions should be deduced only after a careful consideration of them all.

318 JAMES STREET.

